# Pediatric Mixed *Plasmodium vivax*–*P. falciparum* Infection with Disparate Parasitemias: Diagnostic and Surveillance Challenges

**DOI:** 10.3390/children13010145

**Published:** 2026-01-20

**Authors:** Jose Luis Estela-Zape

**Affiliations:** Faculty of Health, Universidad Santiago de Cali, Cali 760035, Colombia; jose.estela00@usc.edu.co or jose.estela55@gmail.com

**Keywords:** *Malaria vivax*, mixed infection, *Malaria falciparum*, glucosephosphate dehydrogenase, antimalarials, malaria

## Abstract

**Highlights:**

**What are the main findings?**

- Mixed *P. vivax*–*P. falciparum* infection with marked parasitemia disparity (5500 parasites/µL *P. vivax* vs. 562 parasites/µL *P. falciparum*) detected using complementary microscopy and rapid diagnostic testing.
- Low-density *P. falciparum* coinfection confirmed through integrated diagnostic approach despite predominant *P. vivax* parasitemia, enabling species-appropriate antimalarial therapy.

**What are the implications of the main findings?**

- Coinfections with markedly disparate parasitemias can be missed by conventional diagnostic methods, posing a risk of delayed or incomplete treatment.
- Comprehensive parasitological evaluation combining microscopy and rapid testing for pan-Plasmodium antigens and HRP2 is recommended in endemic areas to optimize outcomes and prevent severe malaria.

**Abstract:**

**Background and Clinical Significance:** Malaria remains a significant public health issue in Latin America, where *Plasmodium vivax* predominates but *P. falciparum* continues to circulate. Mixed-species infections are uncommon and can pose diagnostic challenges, particularly when parasite densities differ markedly, increasing the risk of underdetecting *P. falciparum* with conventional methods. **Case report:** We report a 9-year-old boy from an endemic area with a six-day febrile syndrome. Thick smear and peripheral blood film microscopy, complemented by rapid diagnostic tests for pan-*Plasmodium* and HRP2 antigens, confirmed a mixed infection with *P. vivax* (5500 parasites/µL) and *P. falciparum* (562 parasites/µL). The patient was hemodynamically stable, without severe malaria criteria, and laboratory values were within normal limits. Following confirmation of normal glucose-6-phosphate dehydrogenase activity, treatment with artemether–lumefantrine was initiated, followed by primaquine for hypnozoite eradication. Clinical evolution was favorable, with progressive defervescence, treatment tolerance, and documented parasite clearance. **Conclusions:** This case illustrates the risk of underestimating *P. falciparum* in mixed infections with disparate parasitemias and highlights the value of integrated diagnostic approaches in resource-limited endemic settings. It also underscores surveillance limitations that can misclassify mixed infections, potentially affecting epidemiological estimates and treatment strategies. Timely recognition and comprehensive diagnostic evaluation are essential to ensure appropriate antimalarial therapy, prevent complications, and inform public health interventions in regions where both species coexist.

## 1. Introduction

Malaria remains a significant public health concern. In 2023, an estimated 263 million cases and approximately 600,000 deaths occurred globally, while the Region of the Americas continued to report active transmission, with more than 537,000 cases notified in 2024 [1,2]. Most cases in the region are concentrated in the Amazon basin and the Pacific coast, with Brazil, Colombia, and Venezuela accounting for the highest burden. In Latin America, *Plasmodium vivax* (*P. vivax*) predominates, responsible for approximately 60–75% of infections, whereas *Plasmodium falciparum* (*P. falciparum*) contributes 25–40%, with geographic variability [3,4].

In Colombia, 127,600 confirmed malaria cases were reported in 2024: *P. vivax* accounted for 62.5%, *P. falciparum* for 35.7%, and mixed infections for 1.8%. Although less frequent, mixed-species infections pose diagnostic challenges, particularly when substantial disparities in parasite density exist, which may limit detection using standard surveillance methods [5,6,7]. In such scenarios, the species present at lower parasitemia levels may be overlooked by conventional microscopy, underscoring the need for complementary diagnostic approaches and careful clinical interpretation.

In endemic settings, diagnosis continues to rely primarily on thick smear microscopy, peripheral blood film, and rapid diagnostic tests. These methods remain the operational foundation of malaria surveillance; however, their sensitivity decreases in low-density infections and mixed-species presentations, which necessitate cautious interpretation within the appropriate epidemiological context [8,9,10,11].

When *P. vivax* and *P. falciparum* coexist with markedly different parasitemia levels, detection of one species, particularly at low density, may be compromised. The sensitivity of thick smear microscopy for parasitemia below 50 to 100 parasites per microliter is notably reduced, and species detection may be lost when one parasite density markedly exceeds the other [5].

Within this framework, we present a pediatric case of mixed *P. vivax* and *P. falciparum* infection with markedly disparate parasitemias, diagnosed using thick smear, peripheral blood film, and rapid diagnostic testing [11]. The objective of this report is to describe a pediatric mixed-species coinfection characterized by substantial differences in parasite density, illustrating the diagnostic challenges that arise when one species circulates at low parasitemia and emphasizing how these constraints affect accurate species identification and surveillance reporting in endemic settings.

## 2. Case Report

A 9-year-old male patient from a malaria-endemic area, with no relevant past medical history, presented with a persistent febrile syndrome of six days’ duration. Fever was objectively documented, with evening spikes reaching a maximum recorded temperature of 38.8 °C, and was associated with chills, holocranial headache, and asthenia. There were no accompanying respiratory, urinary, or gastrointestinal symptoms. The patient reported no recent travel and no family history of malaria. He had routine exposure to mosquitoes in his area of residence and did not regularly use a bed net.

Upon admission, he was hemodynamically stable, with a blood pressure of 94/51 mmHg, heart rate of 96 bpm, respiratory rate of 20 breaths per minute, and oxygen saturation of 95% on room air. Physical examinations showed preserved general condition, normocolored mucosae, absence of jaundice, normal vesicular breath sounds without additional noises, a soft and non-tender abdomen, and a neurological exam without focal deficits or meningeal signs, or clinical evidence of cerebral malaria. No clinical criteria for severe malaria were identified.

No clinical criteria for severe malaria were identified. Initial laboratory studies (Table 1) showed no evidence of severe anemia, significant thrombocytopenia, hepatocellular injury, or renal dysfunction. Anthropometric evaluation revealed a height of 155 cm and weight of 35 kg, with a body mass index appropriate for age.

Table 1 Evolution of clinical laboratory parameters during hospitalization (5 August 2025, 7 August 2025) and outpatient follow-up (22 August 2025). Missing values indicate parameters not repeated per clinical protocol at specified time points.

The diagnosis was established through thick and thin smears interpreted under standardized malaria surveillance protocols (Table 2). A mixed infection with *P. vivax* and *P. falciparum* was confirmed, with quantification performed by trained microscopists. The complementary rapid diagnostic test was positive for pan-*Plasmodium* antigen and HRP2, supporting the confirmation of coinfection, using a combined Pan-*Plasmodium*/HRP2 rapid diagnostic test routinely employed by the local malaria surveillance program. A single combined Pan-*Plasmodium*/HRP2 rapid diagnostic test kit was used in this patient; separate commercial kits for Pan-Plasmodium and HRP2 were not employed.

Parasitological diagnosis confirming mixed *P. vivax*–*P. falciparum* infection. Parasitemia quantified by thick/thin smear microscopy per standardized surveillance protocols. RDT performed with single combined Pan-Plasmodium/HRP2 test kit.

Prior to initiating radical treatment, glucose-6-phosphate dehydrogenase (G6PD) activity was verified and found to be normal, allowing for the safe use of primaquine. Treatment consisted of artemether–lumefantrine (20 mg/120 mg), administered according to the standard regimen: 4 tablets per dose in six scheduled doses at 0, 8, 24, 36, 48, and 60 h, always with fatty foods or milk to optimize absorption. After completion of the full six-dose artemether–lumefantrine regimen, primaquine was initiated for *P. vivax* hypnozoite eradication at 0.25 mg base/kg/day for 14 days. Based on the patient’s weight, approximately 9.25 mg base daily was required; therefore, one 10 mg base tablet per day was prescribed, according to the available formulation. Primaquine was started after the last dose of artemether–lumefantrine, without temporal overlap between both treatments.

During hospitalization days 2 and 3, the patient showed favorable clinical evolution, with progressive defervescence, adequate oral tolerance, hemodynamic stability, and no signs of progression to complicated malaria. Serial clinical assessments and follow-up laboratory tests (Table 1) revealed no significant abnormalities. Parasitological follow-up was performed with thick blood smears every 12 h, with complete parasitemia clearance documented on hospital day 5. The patient was discharged after confirmation of parasitological clearance.

## 3. Discussion

Coinfection with *P. vivax* and *P. falciparum* in children presents diagnostic and therapeutic challenges arising from species-specific biology, differences in parasite density, and variable risk of severe outcomes [12]. This case is consistent with reports indicating that mixed infections with markedly unequal parasitemias can be underdetected, particularly when *P. falciparum* circulates at densities near or below the detection limits of routine microscopy. Low-density *P. falciparum* remains clinically relevant because of its potential for rapid progression when not identified and treated [1,8,13].

The parasitemia disparity in this patient (*P. vivax* 5500 parasites/µL; *P. falciparum* 562 parasites/µL) represents a scenario in which the species associated with higher pathogenicity may occur at concentrations that risk underrecognition. Detection of both species through complementary diagnostic methods supported appropriate therapeutic decision-making and reduced the likelihood of delayed treatment [14,15].

From a pathophysiological perspective, mixed-species malaria involves both independent and overlapping mechanisms. *P. falciparum* can evade host immune responses through antigenic variation, modulation of inflammation, and persistence in microvascular compartments [16,17]. *P. vivax* adds the risk of relapse through hypnozoites and may induce systemic inflammation capable of disrupting hematologic stability [18]. Interspecies interactions have been described to influence immune regulation, hematologic alterations, and intraerythrocytic competition, which may affect overall clinical expression [19].

Although this patient did not exhibit severe manifestations, comprehensive diagnostic evaluation was required. Absence of complications compatible with early intervention, low *P. falciparum* parasitemia (562 parasites/µL), and lack of cerebral malaria criteria despite reported microvascular sequestration risk at low densities [8,20]. Importantly, not all pediatric coinfections progress to severe malaria; previous reports indicate that low parasite density, early diagnosis, and absence of comorbidities can result in benign courses, as observed in this patient.

Clinical reports from pediatric populations in Africa, Asia, and Latin America describe coinfections with variable parasite densities and differing degrees of hematologic compromise. Scalisi et al. (2022) [21], for example, documented a pediatric coinfection complicated by multisystem inflammatory syndrome despite low *P. falciparum* density, illustrating the heterogeneous clinical behavior of mixed infections. Other series also report anemia, thrombocytopenia, and variable therapeutic requirements [22,23]. Compared with these cases, the patient described here exhibited a benign course despite confirmed coinfection.

Mixed-species malaria is associated with higher hematologic risk, including severe anemia from hemolysis or bone marrow suppression, immune-mediated thrombocytopenia, and coagulopathies [10,24,25]. Neurological complications, particularly cerebral malaria due to *P. falciparum*, remain a key concern in endemic regions; however, some pediatric coinfections do not result in severe malaria, as previously described [26].

Therapeutic management prioritized treatment of *P. falciparum* using artemether–lumefantrine, in accordance with international recommendations due to its virulence, risk of recrudescence, and patterns of resistance [15]. In contrast, if a clinician presumes monoinfection with *P. vivax*, treatment may be limited to chloroquine (or other locally recommended anti-*P. vivax* therapy), which would not adequately address the potentially pathogenic low-density *P. falciparum*, underscoring the importance of accurate species identification in mixed infections [15]. This approach aligns with WHO 2025 guidance [27] and the World Malaria Report 2024 [1,28], which recommend treating all mixed infections as *P. falciparum* malaria with artemisinin-based combination therapy administered with fatty food to improve absorption. The regimen was followed by clinical improvement and parasitological clearance, while primaquine was used to eliminate *P. vivax* hypnozoites.

Screening for G6PD deficiency before primaquine initiation is essential to prevent hemolysis. In this patient, G6PD activity was measured quantitatively using a commercially available enzymatic assay (kit not specified to avoid conflict of interest) [29,30], and the absence of deficiency allowed safe administration. Ideally, clinical documentation should specify whether testing was qualitative or quantitative and include hemolysis monitoring between days 3 and 7 of treatment. Current guidelines recommend primaquine 0.25–0.5 mg base/kg/day for 14 days; depending on national policies, a single low dose of 0.25 mg/kg may be used to reduce *P. falciparum* transmission.

The favorable clinical and parasitological response underscores the relevance of timely and comprehensive management of pediatric *P. vivax*–*P. falciparum* coinfections.

### 3.1. Diagnostic Challenges in Mixed-Species Malaria with Disparate Parasitemia

The diagnostic findings in this patient, with *P. vivax* at 5500 parasites/µL and *P. falciparum* at 562 parasites/µL, illustrate the difficulty of detecting low-density coinfections when individual methods are used alone. A *P. falciparum* burden in this range can be missed in thick smear microscopy, particularly when microscopist expertise or examination time is limited, increasing the risk of incomplete diagnosis [12,21,31,32]. Thick/thin smear microscopy combined with HRP2-based rapid diagnostic testing enabled *P. falciparum* detection despite 9.8:1 *P. vivax* predominance, providing diagnostic confirmation without PCR. This integrated approach aligns with the performance characteristics summarized in Table 3 and demonstrates how combining methods with different analytical thresholds enhances diagnostic reliability in endemic settings where mixed infections are common.

Analytical performance compiled from published specifications [8,9,10,11]. Sensitivity reflects parasite density detection limits. RDT refers to HRP2-based rapid diagnostic tests.

An important factor is the masking effect when one species predominates microscopically; high-density *P. vivax* can obscure low-density *P. falciparum*. Systematic examination of thin films improves morphological resolution, while HRP2-positive rapid tests provide timely confirmation of *P. falciparum*, reducing the risk of delayed diagnosis in acute care settings [33,34]. Accurate identification of both species is essential, as misclassifying this case as *P. vivax* monoinfection could have led to incomplete risk assessment and suboptimal therapy [10]. The diagnostic strategy applied here, relying on complementary methods in a context where PCR is not routinely available, supported appropriate therapeutic decision-making and reduced the likelihood of missing a clinically significant coinfection.

### 3.2. Implications for Epidemiological Surveillance and Public Health Policy

This case highlights persistent limitations in malaria surveillance systems across the Amazon basin, particularly concerning the detection of mixed *P. vivax*–*P. falciparum* infections. Although national databases in Colombia report a mixed-infection prevalence of 1.8 percent (2289 of 127,600 cases in 2024), similar values are observed in Brazil, Peru, and Venezuela. However, molecular studies consistently demonstrate substantially higher coinfection rates within the same regions. In the Colombian Amazon, nested PCR identified mixed infections in 43.2 percent of evaluated individuals, predominantly involving *P. vivax* and *P. malariae*. In the Brazilian Amazon, real-time PCR detected 8.0 percent mixed *P. falciparum*–*P. vivax* infections and additional low-frequency combinations. The contrast between surveillance-reported prevalence and molecularly confirmed rates indicates that mixed infections are systematically underestimated rather than uncommon [7,8,35].

This case also illustrates how marked disparities in parasitemia compromise species detection when conventional methods are used in isolation. When one parasite circulates at significantly lower density, microscopy sensitivity decreases, and species may be overlooked. Systematic reviews report that the sensitivity of microscopy for detecting mixed *P. falciparum*–*P. vivax* infections is approximately 45 percent, compared with more than 80 percent for monoinfections. HRP2-based rapid diagnostic tests also show reduced performance at parasitemia levels below 100 parasites per microliter, with further decreases in mixed infections where one species predominates. These diagnostic limitations contribute to systematic underreporting, hinder characterization of transmission patterns, and limit the ability to detect emerging shifts in species distribution or antimalarial resistance [36].

Addressing these gaps requires strengthening diagnostic and reporting practices across endemic regions. Priority actions include implementing dual-confirmation strategies such as microscopy combined with HRP2 rapid tests, or microscopy and PCR where available; establishing standardized training and quality assurance programs to improve diagnostic accuracy and consistency; and modifying surveillance instruments to explicitly document coinfections, parasitemia densities, and species-specific findings. Evidence from molecular analyses in the Peruvian Amazon shows that almost ten percent of *P. falciparum* infections contain multiple parasite clones detectable only through genotyping, reinforcing the need for enhanced diagnostic capacity [20,37,38].

Improved detection and systematic registration of mixed-species infections would contribute to more accurate epidemiological assessments, support refinement of treatment protocols, and inform resource allocation for malaria control. Cross-border surveillance initiatives in Brazil, Venezuela, and adjacent regions demonstrate the value of coordinated monitoring to identify transmission hotspots and guide targeted public health interventions.

### 3.3. Limitations of This Report

This single-case report provides clinically relevant documentation from a resource-limited endemic setting but is bound by certain methodological constraints. Polymerase chain reaction confirmation was not available, reflecting operational characteristics of peripheral health facilities in malaria-endemic regions. Follow-up was restricted to clinical assessment rather than extended parasitological monitoring, which would have allowed verification of sustained parasite clearance. Despite these constraints, the case demonstrates effective diagnosis and management supported by standard diagnostic modalities and evidence-based antimalarial therapy routinely accessible in endemic contexts.

## 4. Conclusions

Pediatric coinfection with *P. vivax* and *P. falciparum* with markedly disparate parasitemias represents an underrecognized diagnostic challenge in endemic regions. Accurate identification of both species using thick and thin smear microscopy combined with rapid diagnostic testing enabled timely, species-specific therapy with artemether–lumefantrine and primaquine following G6PD verification. The patient achieved complete parasite clearance without complications. This case underscores the necessity of comprehensive parasitological evaluation in endemic settings to guide treatment and avoid misclassification, which has implications for clinical management and accurate epidemiological reporting.

## Figures and Tables

**Table 1:** Evolution of clinical laboratory parameters.

Blood Biochemistry Results	Hospitalization		External Control
	5 August 2025	7 August 2025	22 August 2025
Leukocytes, (×10^3^/μL) 4.0–11.0	4.0	5.5	7.8
Neutrophils (%) 42.5–73.2	43.1	67.5	55.9
Lymphocytes (%) 18.0–48.3	44.6	30.6	30.7
Monocytes (%) 2–10	12.0	8.8	8.0
Eosinophils (%) 1–4	0.1	0.2	2.0
Basophils (%) 0–2	0.2	0.2	0.4
Hemoglobin, (g/dL) 11.7–18	11.5	13.3	14.8
Hematocrit, (%) 31.5–50	31.5	34.2	41.0
Platelets, (×10^3^/μL) 150–450	95	140	322
C-reactive protein, (mg/L) 0.8–15.8	18.9		
Sodium, (Na^+^, mmol/L) 135–145	141.2		
Potassium, (K^+^, mmol/L) 3.5–5.1	4.1		
Chloride, (Cl^−^ mmol/L) 97–105	104		
Creatinine, (mg/dL) 0.7–1.3	0.81		
BUN, (mg/dL) 6–20	11.5		
Aspartate aminotransferase, (AST U/L) 5–40	32.38	33.74	28.10
Alanine aminotransferase, (ALT U/L) 7–56	26.77	28.33	24.25
G6PD, (U/g) 7–10	8.5		
Lactic Acid, (mmol/L) 0.5–2.0	0.7		
Glucose, (mg/dL) 60–110	185.48		110
**Arterial blood gas**			
pH 7.35–7.45	7.45		
pCO2, (mmHg) 35–45	30.5		
pO2, (mmHg) 80–100	79.3		
HCO3, (mmol/L) 24–26	23.1		
PaO2/FiO2 > 400	377		
Respiratory Support	Breathing room air		

**Table 2:** Microscopic and rapid diagnostic test findings.

Parameter	Result
Species identified	*P. vivax* and *P. falciparum*
Parasitemia—*P. vivax*	5500 parasites/µL
Parasitemia—*P. falciparum*	562 parasites/µL
Parasitemia ratio	*P. vivax* predominant (≈9.8:1)
RDT—Pan-Plasmodium	Positive
RDT—HRP2	Positive

**Table 3:** Diagnostic performance of methods used for detection of mixed *P. Vivax*–*P. Falciparum* infections.

Diagnostic Method	Sensitivity for *P. vivax* (Parasites/µL)	Sensitivity for *P. falciparum* (Parasites/µL)	Specificity for Mixed-Species Detection	Time to Result	Cost/Feasibility	Limitations
Thick smear microscopy	50–100	50–100	Limited; species with low parasitemia may not be detected	1–2 h	Low cost; HIGH availability	Expert-dependent; misses low-density; time-consuming microscopy.
Thin blood film microscopy	10–50	10–50	Higher specificity; improved species differentiation	1–2 h	Low cost; LIMITED availability	Labor-intensive; requires training; not available rurally.
Rapid diagnostic test—Pan-*Plasmodium* (pLDH)	80–95%	80–95%	Variable; detects multiple species antigens	15–20 min	Low-moderate cost; VERY HIGH availability	Lower sensitivity at low parasite densities; may miss mixed infections with low component.
HRP2-based rapid diagnostic tests	40–80%	93–98%	Variable; HRP2 persistence may affect interpretation	15–20 min	Low-moderate cost; VERY HIGH availability	Reduced sensitivity for non-falciparum; persistent HRP2 antigen post-treatment.
Multiplex PCR/qPCR	<10	<10	High; reliable for detecting mixed infections	8–24 h	High cost, LIMITED availability	Requires infrastructure and training; impractical field-use.

## Data Availability

The authors declare that all data supporting the report are available upon request from the corresponding author.

## Abbreviations

The following abbreviations are used in this manuscript:

WHO	World Health Organization
G6PD	Glucose-6-Phosphate Dehydrogenase
RDT	Rapid Diagnostic Test
AST	Aspartate Aminotransferase
ALT	Alanine Aminotransferase
PCR	Polymerase Chain Reaction
BUN	Blood Urea Nitrogen
PaO2/FiO2	Partial Pressure of Oxygen/Fraction of Inspired Oxygen
ACT	Artemisinin-based Combination Therapy
HRP2	Histidine-rich protein 2
pLDH	Plasmodium lactate dehydrogenase
H	Hours
Min	Minutes
